# Cognitive impairment and levodopa induced dyskinesia in Parkinson’s disease: a longitudinal study from the PACOS cohort

**DOI:** 10.1038/s41598-020-79110-7

**Published:** 2021-01-13

**Authors:** Antonina Luca, Roberto Monastero, Roberta Baschi, Calogero Edoardo Cicero, Giovanni Mostile, Marco Davì, Vincenzo Restivo, Mario Zappia, Alessandra Nicoletti

**Affiliations:** 1 Department of Medical, Surgical Sciences and Advanced Technologies, GF Ingrassia, Neurologic Unit, AOU “Policlinico-Vittorio Emanuele”, University of Catania, Via Santa Sofia n.78, 95100 Catania, Sicily Italy; 2 Department of Biomedicine, Neuroscience and Advanced Diagnostics, University of Palermo, Via G. La Loggia n.1, Palermo, Sicily Italy; 3 Department of Sciences for Health Promotion and Mother-Child Care, University of Palermo, Palermo, Italy

**Keywords:** Neurology, Risk factors

## Abstract

Aim of the study was to evaluate possible associations between cognitive dysfunctions and development of Levodopa Induced Dyskinesia (LID). PD patients from the Parkinson’s disease Cognitive impairment Study cohort who underwent a baseline and follow-up neuropsychological evaluations were enrolled. Mild Cognitive Impairment (PD-MCI) was diagnosed according to MDS level II criteria. The following cognitive domains were evaluated: episodic memory, attention, executive function, visuo-spatial function and language. A domain was considered as *impaired* when the subject scored 2 standard deviation below normality cut-off values in at least one test for each domain. Levodopa equivalent dose, UPDRS-ME and LID were recorded at baseline and follow-up. To identify possible neuropsychological predictors associated with the probability of LID development at follow-up, Cox proportional-hazards regression model was used. Out of 139 PD patients enrolled (87 men, mean age 65.7 ± 9.4), 18 (12.9%) were dyskinetic at baseline. Out of 121 patients non-dyskinetic at baseline, 22 (18.1%) developed LID at follow-up. The impairment of the attention and executive domains strongly predicted the development of LID (HR 4.45;95%CI 1.49–13.23 and HR 3.46; 95%CI 1.26–9.48 respectively). Impairment of the attention and executive domains increased the risk of dyskinesia reflecting the alteration of common cortical network.

## Introduction

Levodopa and dopamine agonists represent the gold-standards for the management of motor symptoms of Parkinson’s disease (PD). Even if their efficacy is undisputed, long-term replacement therapy leads frequently to some motor complications such as wearing-off, on–off fluctuations, dose failure and levodopa-induced dyskinesia (LID), worsening the patient’s quality of life^1^. LID, which includes a variety of involuntary movements ranging from chorea to dystonia, increases with levodopa exposure and affects about 40% of treated PD patients^2^. From a pathophysiological point of view, LID has been related to an overactivation of the motor cortex through the disinhibition of the thalamocortical neurons exerted by levodopa^3^. Moreover, the cortical structures involved in motor program and inhibition such as the supplementary motor area and the inferior frontal cortex have been found to be structurally and functionally impaired in PD patients with LID^3^.

As the name itself suggests, levodopa is necessary but not enough to generate LID. The latter is caused by the pre-synaptic nigro-striatal degeneration which characterizes PD and increases along with disease duration, being associated with a relatively spared post-synaptic nigrostriatal system. This pathological explanation has been supported by data from a longitudinal study, carried out in parkinsonian patients from sub-Saharan region, which showed that LID frequency was more influenced by disease duration and levodopa dose rather than by the timing of levodopa initiation (early or delayed)^4^. Nevertheless, several additional risk factors associated with LID have been reported, including anxiety, rigid-akinetic phenotype, low body mass index, polymorphisms located in the dopamine receptor D2 gene, coffee consumption and female gender^5–8^. Recently, an association between LID and cognitive impairment has been supposed but not fully elucidated^9–11^.

Aim of the present study was to evaluate the presence of possible associations between cognitive impairment and the occurrence of LID. This study is part of The PArkinson’s disease COgnitive impairment Study (PACOS), an observational study involving two Sicilian centers, aimed to assess epidemiologic, clinic and instrumental biomarkers associated with Mild Cognitive Impairment (MCI) in a large hospital-based cohort of PD patients^12–17^.

## Methods

### Study population

PD patients diagnosed according to the Brain Bank criteria^18^, who attended the Neurologic Unit of the “Policlinico Vittorio Emanuele” in Catania and the Memory and Parkinson’s disease Center of the “Policlinico Paolo Giaccone” in Palermo, were enrolled in the PACOS cohort, which includes 659 non-demented PD patients at baseline. As previously reported^14^, we selected all PD patients (n = 139) who underwent at least two comprehensive neuropsychological evaluations (baseline and follow-up) during a period of maximum 48 months (between 12 and 48 months). Background and methods have been described elsewhere in details^12–14^.

All participants provided written informed consent prior to be enrolled in the study, which was approved by Local Ethical Committee of the University Hospital of Palermo, P. Giaccone (approval number: 14:03/2018) and was in accordance with the Declaration of Helsinki.

### Clinical and neuropsychological assessment

At baseline and at follow-up, all the enrolled patients were evaluated by movement disorders specialists with a standard neurological examination. Demographic, clinical and pharmacological data were collected from patient’s medical records. PD severity was evaluated in “off” state with the Unified Parkinson Disease Rating Scale – Motor Examination (UPDRS-ME) and the Hoehn and Yahr (HY) scale. Levodopa Equivalent Daily Dosage (LED)^19^ was calculated both at baseline and at follow-up evaluations. The presence of dyskinesia was defined at baseline and at follow-up according to the item 32 of the UPDRS section IV. PD patients were divided into patients without LID (LID-) and those with LID (LID +). At baseline patients LID + were classified as having troublesome dyskinesias according to a score ≥ 2 of the item 33 of the UPDRS section IV.

All PD subjects underwent a comprehensive neuropsychological and behavioral assessment when in “on” state. Neuropsychological evaluations were performed by neurologists with a specific expertise in neuropsychology and dementia, and the same rater performed both baseline and follow-up assessments.

According to MDS level II criteria, the following five cognitive domains were evaluated with two tests for domain: episodic memory (Rey’s Auditory Verbal Learning Test and Prose recall test with a delayed recall condition); attention (Stroop color-word test and Trail Making Test part A); executive functioning (Verbal fluency letter test and Colored Raven’s Progressive Matrices); the visuo-spatial functioning (Clock drawing test and Copy of figures); language (Aachener Aphasie Test-Naming item and the short version of the Token test). A domain was considered as “impaired” when the patient scored 2 standard deviations below normality cut-off values in at least one test in the specific domain, regardless the presence of MCI. Diagnosis of PD-MCI was made according to the Movement Disorder Society Task Force criteria-level II^20^. Diagnosis of PDD was made according to the MDS criteria^21^.

Details about the neuropsychological assessment performed in the PACOS cohort have been extensively reported elsewhere^12–14^.

### Statistical analysis

Data were analysed using STATA 12.1 software packages (StataCorp. 2011. Stata Statistical Software: Release 12. College Station, TX: StataCorp LP). Data cleaning was performed before the data analysis considering both range and consistence checks. Quantitative variables were described using mean and standard deviation. The difference between means and proportions was evaluated by the t-test and the Chi square test, respectively. In case of a not-normal distribution, appropriate non-parametric tests were performed.

A first analysis was carried out considering all the enrolled patients at baseline. We also performed two different time to event analysis. From the one hand, we evaluated the role of the impairment in a specific cognitive domain at baseline and the risk of LID at follow-up (outcome variable) and, on the other hand, we have also evaluated the relationship between the presence of LID at baseline and the risk of cognitive impairment (PD-MCI and PDD) at follow-up. Cox proportional-hazards regression model was used for both the univariate and multivariate analyses. Variables with p value < 0.1 at univariate analysis were included in the final multivariate Cox models. Schoenfeld residuals test was used for testing the proportional hazard. 95% confidence interval (CI), and p value (two-tailed test, a = 0.05) were calculated. Whenever variables were dichotomized or polychotomized, the cut-offs were derived from the pooled distribution of case and control subjects (e.g., using the median value).

## Results

### Baseline characteristics of the sample

As previously reported, 139 PD patients (87 men, 62.6%) with a mean age of 65.7 ± 9.4 and a mean disease duration of 3.0 ± 2.8 years who underwent at least two neuropsychological evaluations between 12 and 48 months were enrolled in the present study. The mean UPDRS-ME score was 26.2 ± 13.5 and the mean Hoehn and Yahr stage was 2.0 ± 0.7. At baseline, 84 (60.4%) were PD with normal cognition (PD-NC), while 55 (39.6%) fulfilled the MDS-level II diagnostic criteria for PD-MCI. Of the 139 PD patients at baseline 18 (12.9%) presented LID (Table 1) and among them 11 (61.1%) had troublesome LID. At baseline, depending on the presence of at least one test altered in a specific domain, independently from the presence of MCI or not, the memory domain was impaired in 41 (29.5%) PD patients, the executive functioning domain in 40 (28.8%) , the visuo-perceptual domain in 23 (16.5%), the attention domain in 40 (28.8%), and the language domain was impaired in 2 (1.4%) PD patients.

**Table 1:** Demographic and clinical characteristics at baseline.

	LID- N = 121	LID + N = 18	p value
**Male, n (%)**	77 (63.6)	10 (55.6)	0.5
**Age, years**	66.3 ± 9.1	61.9 ± 10.9	0.07
**Age at onset, years**	63.7 ± 9.4	56.3 ± 11.8	**0.005**
**Disease duration, years**	2.6 ± 2.5	5.7 ± 3.2	** < 0.0001**
**UPDRS-ME score**	24.7 ± 12.5	36.0 ± 14.8	**0.002**
**LED mg/day**	266.5 ± 285.9	935.6 ± 443.5	** < 0.0001**
LED (< 300 mg)	86 (71.1)	2 (11.1)	**/**
LED (> 300 mg)	35 (28.9)	16 (88.9)	** < 0.0001**
**Education, years**	8.7 ± 4.6	10.7 ± 4.1	0.08
**Presence of MCI**	46 (38.0%)	9 (50%)	0.3
**Impaired domains**			
Episodic memory	34 (28.1%)	7 (38.9%)	0.3
Executive functioning	30 (24.8%)	10 (55.6%)	**0.01**
Attention	36 (29.7%)	4 (22.2%)	0.5
Visuo-spatial function	21 (17.4%)	2 (11.1%)	0.5
Language	1 (0.8%)	1 (5.6%)	0.2

LID: levodopa induced dyskinesia; UPDRS-ME: Unified Parkinson’s Disease Rating Scale-Motor Examination; LED: levodopa equivalent dosage; MCI: mild cognitive impairment.

### Cognitive impairment and risk of LID

Out of the 121 patients without LID at baseline, 22 (18.1%) developed LID at follow-up (LID +); the mean follow-up time was 24.0 ± 10.2 months. At univariate Cox proportional hazard regression models, disease duration, UPDRS-ME, LED at baseline, education and the impaired attentive domain were significantly associated with the development of LID, as reported in Table 2.

**Table 2:** Clinical characteristics at baseline and risk of Levodopa induced dyskinesia at follow-up.

	LID- N = 99	LID + N = 22	Univariate analysis		
			HR	95%CI	p value
**Male, n (%)**	63 (63.6)	14 (63.6)	1.02	0.43–2.58	0.9
**Age, years**	66.3 ± 9.3	66.3 ± 8.3	1.01	0.96–1.07	0.5
**Age at onset, years**	63.9 ± 9.4	62.8 ± 9.4	1.00	0.95–1.05	0.9
**Disease duration, years**	2.4 ± 2.3	3.5 ± 3.3	1.14	0.99–1.30	0.06
**UPDRS-ME score**	22.9 ± 11.0	32.9 ± 15.7	1.04	1.01–1.07	**0002**
**LED mg/day (baseline)**	210.9 ± 195.1	548.9 ± 430.3	1.002	1.001 ± 1.003	** < 0.0001**
LED (< 300 mg)	78 (78.8)	6 (27.3)	1	/	/
LED (> 300 mg)	21 (21.2)	16 (72.7)	10.1	3.37–30.05	** < 0.0001**
**LED mg/day (follow-up)**	499.6 ± 348.5	672.9 ± 588.2	1.0007	1.000–1002	0.1
LED (< 500 mg)	63 (63.6)	13 (59.1)	1	/	
LED (> 500 mg)	36 (36.4)	9 (40.9)	1.18	0.49–2.81	0.7
**Education, year**	9.2 ± 4.6	6.2 ± 3.5	0.80	0.70–0.91	**0.001**
**Presence of MCI**	34 (34.3)	12 (54.5)	1.39	0.59–3.29	0.4
**Impaired domains at baseline**					
Episodic memory	28 (28.3)	6 (27.2)	0.50	0.18–1.41	0.2
Executive functioning	20 (20.2)	10 (45.4)	1.99	0.85–4.64	0.1
Attention	26 (26.3)	10 (45.4)	2.67	1.07–6.66	**0.03**
Visuo-spatial function	15 (15.1)	6 (27.3)	2.08	0.78–5.50	0.1
Language	1 (1.0)	0	/	/	/

LID: levodopa induced dyskinesia; UPDRS-ME: Unified Parkinson’s Disease Rating Scale-Motor Examination; LED: levodopa equivalent dosage; MCI: mild cognitive impairment.

At multivariate analysis, adjusting by age and sex considered *as *a priori confounders, UPDRS-ME, LED at baseline and education predicted the development of LID, while the impaired executive functioning domain at baseline was borderline significantly associated with the development of LID (HR 2.45; 95%CI 0.89–6.71*; p value* 0.08). However, when analysis was adjusted by LED at follow-up, rather than at baseline, a stronger and significant association between the impaired executive functioning domain at baseline and the development of LID has been found (HR 3.46; 95%CI 1.26–9.48; p value 0.02) (Supplemental Table 1).

On the other hand, at multivariate analysis (adjusting by age, sex, UPDRS-ME, LED at baseline and education) the presence of an impaired attentive domain at baseline strongly predicted the development of LID (HR 4.69; 95%CI 1.40–15.70; p value 0.01). A close association was also found when the analysis was adjusted by LED at follow-up (HR 4.45; 95%CI 1.49–13.23; p value 0.007) (Supplemental Table 2).

In the multivariate analysis concerning the relationship between development of LID and presence of an altered visuo-spatial domain at baseline, no association was found in both the models with LED at baseline or LED at follow-up.

### LID and risk of cognitive impairment

Out of the 84 PD-NC at baseline, 28 (33.3%) developed PD-MCI while 4 (4.8%) developed PDD at follow-up. The possible relationship between the presence of LID at baseline and the development of MCI at follow-up has been evaluated. At univariate analysis, only age and education were significantly associated with the development of PD-MCI. However, at multivariate analysis Cox proportional hazard (adjusting by age, sex, UPDRS-ME, education and LED at follow-up), the presence of LID at baseline was strongly associated with the development of PD-MCI even if such association was borderline significant (HR 4.98; 95%CI 0.93–26.61; p *value* 0.06) (see Table 3). At baseline out of the 9 PD-NC LID + patients, 7 had troublesome LID and of these 3 (42.9%) developed MCI.

**Table 3:** Predictors of mild cognitive impairment at follow-up.

	PD-NC (52)	PD-MCI (28)	Univariate analysis			Multivariate analysis		
			HR	95%CI	p value	HR	95%CI	p value
Sex (M)	33 (63.4%)	18 (64.3)	1.30	0.58–2.90	0.5	0.93	0.39–2.27	0.9
Age, years	62.2 ± 10.2	68.8 ± 9.8	1.04	1.002–1.09	**0.04**	1.04	0.99–1.10	0.09
UPDRS-ME	24.6 ± 14.0	24.5 ± 13.0	0.98	0.95–1.01	0.2	0.97	0.94–1.007	0.1
LED at follow-up	643.5 ± 506.4	632.0 ± 446.4	1.00	1.00–1.006	0.5	1.00	0.998–1.0007	0.4
Education, years	10.6 ± 4.1	7.5 ± 4.8	0.90	0.82–0.98	**0.02**	0.90	0.82–1.00	**0.04**
LID at baseline	5 (9.6)	3 (10.7)	0.99	0.29–3.31	0.9	4.98	0.93–26.61	0.06

PD-NC: Parkinson’s disease-normal cognition; PD-MCI: Parkinson’s disease-mild cognitive impairment; M: male; UPDRS-ME: Unified Parkinson’s Disease Rating Scale-Motor Examination; LED: levodopa equivalent dosage; LID: levodopa induced dyskinesia.

Considering the risk of PDD, out of the 139 non-demented patients at baseline, 18 (12.9%) developed PDD at follow-up. At univariate analysis, age, UPDRS-ME, education and the presence of PD-MCI at baseline were significantly associated with the development of PDD. However, at multivariate analysis (adjusting by LED at follow-up, sex, age, education, UPDRS-ME and the presence of PD-MCI), the presence of LID at baseline predicted the development of PDD at follow-up (HR 5.58; 95%CI 0.88–35.38; p *value* 0.06) even if also in this case such association was borderline significant, as shown in Table 4. At baseline out of the 18 LID + patients, 11 (61.1%) had troublesome LID and of these, 2 (18.2%) developed PDD.

**Table 4:** Predictors of Parkinson’s disease-dementia at follow-up.

	PD-NC (121)	PDD (18)	Univariate analysis			Multivariate analysis		
			HR	95%CI	p value	HR	95%CI	p value
Sex (M)	75 (62.0)	12 (66.7)	1.21	0.43–3.37	0.7	4.80	1.21–19.01	**0.02**
Age, years	65.3 ± 9.5	68.3 ± 8.4	1.07	1.004–1.151	**0.03**	1.06	0.98–1.14	0.1
UPDRS-ME score	25.1 ± 12.6	33.1 ± 16.5	1.03	1.00–1.05	0.06	1.03	0.99–1.08	0.09
LED at follow-up	605.0 ± 458.5	603.5 ± 447.9	1.00	1.00–1.001	0.8	1.00	0.998–1.001	0.7
Education, years	9.2 ± 4.5	8.9 ± 4.6	0.88	0.78–0.99	**0.04**	0.83	0.70–0.97	**0.02**
LID at baseline	15 (12.4)	3 (16.7)	1.85	0.52–6.56	0.3	5.58	0.88–35.38	**0.07**
PD-MCI	41(33.9)	14 (77.8)	4.37	1.41–13.50	**0.01**	8.94	2.07–38.57	**0.003**

PD-NC: Parkinson’s disease-normal cognition; PDD: Parkinson’s disease-dementia; M: men; UPDRS-ME: Unified Parkinson’s Disease Rating Scale-Motor Examination; LED: levodopa equivalent dosage; LID: levodopa induced dyskinesia; PD-MCI: Parkinson’s disease-mild cognitive impairment.

## Discussion

Our study suggests a bidirectional relationship between LID and cognitive impairment in PD. In particular, the impairment of executive and attentive functioning increased the risk of future LID development in PD, while the presence of LID at baseline predicts the risk of cognitive impairment.

LID represents a source of distress for PD patients and negatively affects patients’ quality of life and influences treatment decisions^22^. The identification of those patients at greater risk of dyskinesia may result in a better clinical approach. Therefore, the interest in evaluating possible predictors of LID is growing. The existence of an association between LID and cognitive decline is still controversial^9,10^. Although LID has recently been proposed as risk factor for cognitive decline^11^, the role of cognitive impairment in LID occurrence has been scarcely investigated.

In particular, two recent longitudinal studies on early PD patients did not find any association between the presence of cognitive impairment and LID occurrence^9,10^. Similarly, a recent study on PDD and patients with Lewy Bodies Disease did not report any association between cognitive impairment and LID occurrence^23^. However, to the best of our knowledge this is the first longitudinal study assessing the association between the presence of cognitive impairment, diagnosed according the currently accepted MDS Level II diagnostic criteria^20^ and LID occurrence.

In agreement with previous studies^9,10^, LID+ patients had a younger age at onset, a longer disease duration, a more severe motor impairment and took higher levodopa dosages at baseline. Differently for the present data, Yoo et al.^11^ did not find any difference in neuropsychological performances (including those evaluating executive functioning) at baseline when comparing PD patients with LID at follow-up and PD patients without LID at follow-up. Executive functioning (i.e. working memory, flexible thinking, planning, inhibitory control, set-shifting, decision-making tasks) and attention are cognitive abilities needful for everyday decision-making and frequently related to the dopaminergic fronto-striatal network^24,25^.

### Cognitive impairment and risk of LID

Out of 121 patients LID- at baseline, 18.1% developed LID at follow-up. At multivariate analysis, the presence of an impairment in executive functioning and attention domains at baseline was strongly associated with LID appearance, with HR of 3.46 and 4.64 (adjusting for age, sex, UPDRS-ME, education and LED at follow-up) respectively. Contrarily to what previously reported^9,10^, in the PACOS cohort the presence of cognitive impairment increased the risk of LID occurrence. However, it should be noted that while Kelly and coll. and Euseby and coll. assessed cognitive abilities using only a test of global cognition, in the present study the use of a “comprehensive” neuropsychological battery for PD-MCI allowed us to explore possible associations between the impairment of specific cognitive domains and LID.

### LID and risk of cognitive impairment

Considering the 84 PD-NC at baseline 28 (33.3%) developed PD-MCI while 4 developed PDD (4.8%), while regarding the whole sample of 139 non-demented PD patients, 12.9% developed PDD at follow-up^14^. PD-MCI was strongly associated with old age and low education level, whereas—and as previously detailed^14^—the stronger predictor of PDD occurrence was the presence of PD-MCI at baseline. In addition to these reported risk factors, in this study we found a strong positive association, even if borderline significant, between the presence of LID at baseline and the risk of both PD-MCI (HR 4.98; p value 0.06) and PDD (HR 5.58; p value 0.07) at follow-up. Despite methodological and study design differences, these findings are in agreement with the longitudinal study carried out by Yoo et al., in which the presence of LID increased almost four times the risk of conversion to PDD with accelerated executive functioning and global cognitive decline^11^. Similarly, Zhu et al.^26^ performed a longitudinal study reporting a mild association between the presence of dyskinesia at baseline and the development of PDD after five years after univariate analysis (HR 1.18); however, the reported association was no longer significant after multivariate analysis.

Although cognitive impairment and dyskinesia represent frequent complications of PD, only few longitudinal studies to date have evaluated the association between these two phenomena. To the best of our knowledge, the present study represents the first report of an association between the impairment of the executive and attentive functioning domains and LID development in PD. The latter association could be related to the dysregulation of common cortical network. Indeed, it has been supposed that in PD the decreased inhibitory action of the prefrontal cortex on the motor cortex could produce a disinhibition in both motor (involuntary movements) and cognitive control loops (loss of inhibitory, self-regulated and goal directed behaviors).

The impairment of attentive and executive functioning has been strictly associated with prefrontal cortex dysfunction in PD, due to the striatal dopaminergic degeneration which negatively affects the fronto-striatal circuitry in the course of the disease^25^. This hypothesis is supported by data from a recent neuropsychological and behavioral study in which the authors evaluated the ability to *self-regulate* in patients with PD, reporting higher impulsivity score and lower inhibitory control in PD patients with LID than those without^27^. Similarly, in a computerized cognitive study using a multiple object tracking paradigm, PD patients even in early stages showed an impairment of visuospatial sustained attention, resulting in a difficulty in ignoring *non-significant stimuli*^28^.

Actually, the frontal lobe dysfunction in dyskinetic PD patients has been also demonstrated by the reported reduction of the connectivity between the right inferior frontal cortex and the left motor cortex^29^ as well as the greater volume of the right inferior frontal cortex^30^.

To deepen the knowledge related to the possible association between cognitive and motor control in PD appears to be of considerable interest not only because both phenomena represent a source of distress for PD patients, but also for the possible link between the impairment of executive and attentive dysfunction and some *disinhibitory disorders* (i.e. impulse control disorders) belonging, as well as dyskinesia, to anti-parkinsonian drugs related side effects^31^.

To the best of our knowledge, this is the first longitudinal study assessing the association between the presence of cognitive impairment, diagnosed according to the current MDS Level II diagnostic criteria^20^ and LID occurrence in PD. Nevertheless, some limitations should be taken into account when interpreting these findings. First, patients were not evaluated with the same follow-up length, which ranged from 12 to 48 months. Thus, this variability did not exclude that the absence of dyskinesia in some patients may be due to the shorter duration of follow-up. Second, even though the presence of troublesome LID has been evaluated, due to the small number of events a statistical analysis evaluating the impact of the severity of dyskinesia on future development of cognitive impairment could not be performed. Third, caution is needed in extending these findings to the general population because the study sample was recruited in a specialized clinical setting, with inherent possibility of selection bias. Lastly, although analyses were adjusted for major potential confounders, residual confounding cannot be excluded (ie. anxiety and depression, low body mass index at baseline).

However, considering that treating LID or preventing its appearance is possible, our findings may have relevant prognostic and therapeutic implications. Larger prospective cohort studies, with quantitative and qualitative assessment of LID and fixed follow-up time are required to confirm the interplay between LID and cognitive impairment in PD.

### Ethic approval

The study was approved by the local medical Ethics Committee and was in accordance with the Declaration of Helsinki.

## Supplementary Information

Supplementary Tables.

## Data Availability

Anonymized data will be shared by request.
