# Supplementary material for: Cognitive impairment and levodopa induced dyskinesia in Parkinson’s disease: a longitudinal study from the PACOS cohort
Source: Sci Rep. 2021 Jan 13;11:867. doi: 10.1038/s41598-020-79110-7 (PMC7806828; doi:10.1038/s41598-020-79110-7)
Supplement: Supplementary file 1 — Supplementary Tables. [file 41598_2020_79110_MOESM1_ESM.doc]

**Cognitive impairment and levodopa induced dyskinesia in Parkinson’s disease. A longitudinal study from the PACOS cohort.**

**Antonina Luca, MD, PhD, Msc, Roberto Monastero, MD, PhD, Roberta Baschi, MD, Calogero Edoardo Cicero, MD, Msc, Giovanni Mostile, MD, PhD, Marco Davì, MD, Vincenzo Restivo, MD, Mario Zappia, MD, and Alessandra Nicoletti, MD, Msc.**

**Supplemental table** 1. Impairment in executive functioning domain and risk of Levodopa induced dyskinesia

| **Model 1** | **HR** | **95%CI** | **p-value** | **Model 2** | **HR** | **95%CI** | **p-value** |
| --- | --- | --- | --- | --- | --- | --- | --- |
| **Sex (F)** | 0.78 | 0.28-2.20 | 0.6 | **Sex (F)** | 0.78 | 0.27-2.23 | 0.6 |
| **Age, years** | 0.96 | 0.89-1.05 | 0.4 | **Age, years** | 0.96 | 0.89-1.03 | 0.2 |
| **UPDRS-ME score** | 1.02 | 0.98-1.06 | 0.4 | **UPDRS-ME score** | 1.04 | 1.006-1.07 | **0.02** |
| **LED at baseline** | 1.002 | 1.000-1.003 | **0.003** | **LED at follow-up** | 1.0008 | 0.999-1.001 | 0.1 |
| **Education, years** | 0.79 | 0.68-0.93 | **0.003** | **Education, years** | 0.77 | 0.66-0.90 | **0.001** |
| **Executive functioning impaired** | 2.45 | 0.89-6.71 | **0.08** | **Executive functioning impaired** | 3.46 | 1.26-9.48 | **0.02** |

**Legend:** Multivariate analysis (Cox proportional hazard). Model 1 adjusting by LED at baseline; Model 2 adjusting by LED at follow-up. F: female; UPDRS-ME: Unified Parkinson’s Disease Rating Scale-Motor Examination; LED: Levodopa Equivalent Dosage.

**Supplemental table 2. Impairment in attention domain and risk of Levodopa induced dyskinesia**

| **Model 1** | **HR** | **95%CI** | **p-value** | **Model 2** | **HR** | **95%CI** | **p-value** |
| --- | --- | --- | --- | --- | --- | --- | --- |
| **Sex (F)** | 0.49 | 0.16-1.51 | 0.2 | **Sex (F)** | 0.48 | 0.16-1.40 | 0.2 |
| **Age, years** | 0.95 | 0.88-1.04 | 0.2 | **Age, years** | 0.95 | 0.88-1.03 | 0.2 |
| **UPDRS-ME score** | 1.02 | 0.99-1.00 | 0.2 | **UPDRS-ME** | 1.05 | 1.01-1.08 | **0.004** |
| **LED at baseline** | 1.002 | 1.001-1.003 | **0.002** | **LED at follow-up** | 1.000 | 0.999-1.001 | 0.4 |
| **Education, years** | 0.74 | 0.61-0.91 | **0.003** | **Education, years** | 0.74 | 0.61-0.89 | **0.001** |
| **Attention impaired** | 4.69 | 1.40-15.70 | **0.01** | **Attention impaired** | 4.45 | 1.49-13.23 | **0.007** |

**Legend:** Multivariate analysis (Cox proportional hazard). Model 1 adjusting by LED at baseline; Model 2 adjusting by LED at follow-up. F: female; UPDRS-ME: Unified Parkinson’s Disease Rating Scale-Motor Examination; LED: Levodopa Equivalent Dosage
